# In situ scanning gate imaging of individual quantum two-level system defects in live superconducting circuits

**DOI:** 10.1126/sciadv.adt8586

**Published:** 2025-04-30

**Authors:** Marius Hegedüs, Riju Banerjee, Andrew Hutcheson, Tomas Barker, Sumedh Mahashabde, Andrey V. Danilov, Sergey E. Kubatkin, Vladimir Antonov, Sebastian E. de Graaf

**Affiliations:** ^1^ National Physical Laboratory, Teddington TW11 0LW, UK.; ^2^ Physics Department, Royal Holloway University of London, Egham, UK.; ^3^ Department of Microtechnology and Nanoscience MC2, Chalmers University of Technology, SE-412 96 Göteborg, Sweden.

## Abstract

The low-temperature physics of structurally amorphous materials is governed by low-energy two-level system (TLS) defects. Being impervious to most traditional condensed matter probes, the exact origin and nature of TLS remain elusive. Recent advances toward realizing stable high-coherence quantum computing platforms have increased the importance of studying TLS in solid-state quantum circuits, as they are a persistent source of decoherence and instability. Here, performing scanning gate microscopy on a live superconducting NbN resonator at millikelvin temperatures, we locate individual TLS, directly revealing their microscopic nature. Mapping and visualizing the most detrimental TLS in the bath pinpoints the dominant sources of ubiquitous 1/*f* dielectric noise and energy relaxation. We also deduce the three-dimensional orientation of individual TLS electric dipole moments. Combining these insights with structural information of the underlying materials can help unravel the detailed microscopic nature and chemical origin of TLS, directing targeted strategies for their eventual mitigation.

## Teaser

Scanning probe microscope locates and studies individual loss and decoherence sources in live superconducting circuits.

## INTRODUCTION

Glassy disordered materials show remarkable universality in their low-temperature thermal, acoustic, and microwave absorption properties, irrespective of their chemical compositions (*1*, *2*). To explain these intriguing observations, Anderson *et al.* (*3*) and Phillips (*4*) proposed the microscopic model of these materials to be dominated by low-energy imperfections, namely, two-level system (TLS) defects, with their collective behavior described by the phenomenological standard tunneling model (*5*). However, in the half century since, the direct probing of individual TLS defects and testing the microscopic principles underlying these macroscopic observations have been very challenging, fuelling intense debates over the exact nature of these defects (*2*, *6*).

Although TLS have been extensively studied in glassy materials, recent advances in quantum computation and sensing have further underscored the need to characterize their chemical and structural properties (*6*, *7*). TLS are a major source of noise and decoherence, and even a single defect can spoil the performance of an entire circuit (*6*, *8*, *9*). Achieving fault tolerant quantum computing requires stable, high coherence qubits, which in turn need new tools to find, characterize, and understand the nature of TLS defects as they appear in live circuits. This task has been particularly challenging, as the low-energy scales of the defects render them inaccessible to much of conventional material science techniques (*7*).

Established in-operando methods of detecting TLS—for example, by tuning a qubit into resonance with it (*6*, *10*) or similarly tuning TLS by applying strain (*11*) or electric (*7*, *12*–*18*) fields—are incapable of directly extracting precise defect locations. Furthermore, they only indirectly suggest the microscopic origin, revealing no chemical or structural information. On the other hand, traditional scanning probe techniques such as scanning tunneling microscopy or atomic force microscopy (AFM) are routinely used for defect characterization and offer atomic-scale spatial resolution. Unfortunately, these techniques fall short by orders of magnitude in resolving the typical TLS energy scale. Despite this, scanning probe imaging of quantum circuits is becoming increasingly important for characterizing electromagnetic field distributions and material properties in quantum devices at low temperatures (*19*–*25*).

Here, we integrate scanning gate microscopy (SGM) with in situ readout of live superconducting quantum circuits at millikelvin temperatures to locate individual TLS defects, directly demonstrating their microscopic nature. The SGM can also operate in AFM mode for imaging device topography to correlate defect locations with device features and materials. The technique is also able to deduce the electric dipole moment orientation of individual TLS, information that has remained elusive since they were conceptually put forward over half a century ago. By combining information about TLS orientation with detailed material structure and ab initio calculations, our approach could help identify the origin and physical nature of these defects, leading to better solid state quantum circuits.

## RESULTS

### SGM experimental setup and methodology

**Fig. 1.**
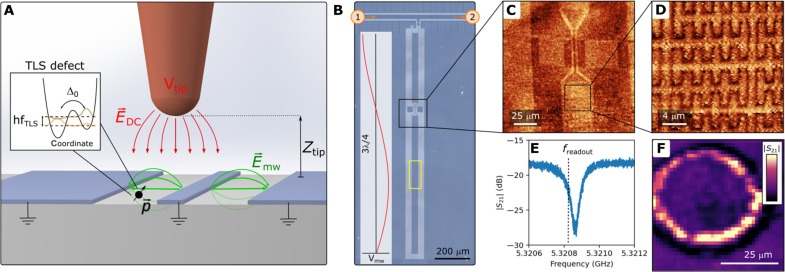
Experimental setup for imaging TLS. (**A**) Schematic of our setup shows a sharp tip above a live circuit in which TLS defects reside. The TLS dipole moment $\vec{p}$ couples to the circuit via the microwave electric field ${\vec{E}}_{\text{mw}}$. The tip is used for both AFM imaging and applying localized electric fields ${\vec{E}}_{\text{DC}}$. (**B**) Optical image of the 3λ/4 superconducting hanger resonator patterned in 40-nm–thick NbN film on sapphire used in our study. For more details on the sample layout, see the Supplementary Materials. The yellow rectangle indicates the location of the scan presented in Fig. 2. The inset shows the microwave voltage amplitude along the resonator. (**C**) Wide-area topographic AFM images over the live circuit taken in the region indicated by the black box in (B). The images are taken by running a proportional–integral–derivative (PID) loop maintaining a constant frequency shift by varying the tip-sample distance. (**D**) Higher-resolution AFM image taken at 200 mK shows the interdigitated capacitors within the resonator. (**E**) Example of the microwave transmission *S*_21_(*f*), measured between the ports marked 1 and 2 in (B), around the circuit resonance frequency *f*_res_. To detect TLS while scanning, we use a heterodyne readout scheme that measures the transmission at *f*_readout_, slightly detuned from *f*_res_. (**F**) Detecting a TLS. The *S*_21_(*f*_readout_) transmission signal [color scale in arb. units (a.u.)] as a function of tip position reveals a bright contour corresponding to a constant electric field from the tip at the location of a TLS in the center. The data were taken with *Z*_tip_ = 15 μm and *V*_tip_ = −10.25 V. See text for further details.

Our experimental setup combining SGM with in situ device readout is described in the schematic presented in Fig. 1A. We use an electrochemically etched tungsten tip attached to a quartz tuning fork to facilitate AFM imaging. The tip is also connected to a voltage source for applying local electric fields to tune the energy of TLS. The entire setup is enclosed within a light-tight, magnetically shielded volume and suspended on springs below the mixing chamber plate of a dry dilution refrigerator to minimize vibrations. With this setup, we achieve a sample stage temperature of ∼45 mK, as measured using a calibrated RuO_*x*_ thermometer.

Although our SGM setup is entirely agnostic to the circuit under study and is adaptable for examining any quantum component coupled to TLS, here, we choose to study a superconducting resonator. Our sample consists of a 3λ/4 resonator patterned in 40-nm–thick NbN on sapphire, an optical image of which is shown in Fig. 1B. Interdigitated capacitors concentrate the electric fields inside the resonator, enabling enhanced coupling to a large number of TLS defects. Further details about theresonator sample and its design can be found in (*26*, *27*) and in the Supplementary Materials.

Figure 1 (C and D) shows AFM images of the live resonator obtained at 200 mK. This in situ AFM imaging enables us to locate various device features. In all our experiments, we limit the scan speed to reduce heating of the piezo positioners and keep the sample temperature below 300 mK at all times, avoiding TLS saturation occurring for *k*_B_*T* > 2*hf*_res_ (*28*) or TLS bath reconfiguration (*29*).

### Detection of TLS defects by SGM

Following AFM imaging, we locate individual TLS as follows. We position the tip at a constant height *Z*_tip_ above the sample and at each grid point in the *xy*-plane conduct a tip voltage sweep. We continuously, in operation, monitor the microwave signal transmitted at a fixed frequency *f*_readout_ slightly offset from the resonance frequency *f*_res_ of the imaged device using a heterodyne detection measurement scheme (see the Supplementary Materials for details). A change in the measured signal *S*_21_(*f*_readout_) thus means that either the resonator’s center frequency or quality factor has changed, the result of a TLS becoming resonant with the resonator. The microwave power level used for transmission measurements was kept very low to avoid saturating TLS, typically with an average photon population of 10 to 1000 in the resonator. A slice of such a dataset *S*_21_(*x*, *y*, *V*_tip_ = const) is shown in Fig. 1F, where data from a 40 by 40 pixel grid are presented. The observed ring indicates a locus of points at which the tip needs to be positioned for the TLS to experience the same electric field magnitude along its dipole moment orientation, such as to bring it into resonance with the resonator. This means that the TLS is located at the center of the ring. On the basis of the location of this grid above the resonator, this defect is thus located within the gap of the interdigitated capacitor of the resonator (see Fig. 1D), this is where we expect the strongest microwave electric fields in the resonator and the strongest TLS-resonator coupling. In the Supplementary Materials, we show more data taken at high microwave powers that confirms the saturation of the detected TLS.

**Fig. 2.**
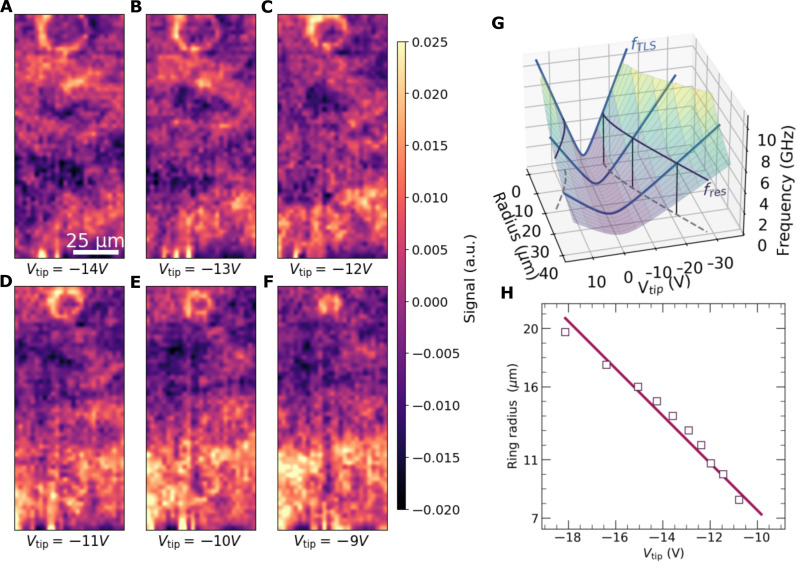
Tuning TLS with SGM. (**A** to **F**) Voltage slices taken with the tip 20 μm above the sample show a bright, circular TLS ring near the top, along with local fluctuations in the plotted *S*_21_ signal. Changing *V*_tip_ changes the size of the circular contour. The scans are taken in a 27 by 60 pixel grid over a 67.5 μm–by–150 μm region marked by the yellow box in Fig. 1B, containing the interdigitated planar capacitor of the resonator as shown in Fig. 1D. The scale bar in (A) is shared across (A) to (F). The color scale shows the change in measured signal *S*_21_ in arbitrary units. (**G**) Conceptual sketch showing the relationship between tip voltage and ring radius for a resonant TLS. Sweeping the tip voltage alters the TLS energy, according to Eq. 1 (blue curves). TLS hyperbolas intersect *f*_res_ (black curves), at which point, the TLS is detected by the resonator as a change in transmission *S*_21_. (**H**) Radius of the ring in (A) to (F) (markers) decreases with increasing tip voltage. The solid line is a linear fit. See movie S1 for the full dataset.

Varying the voltage at which the two-dimensional slice is taken changes the ring diameter, as shown in Fig. 2 (A to F) (for a different TLS than in Fig. 1F). These data were taken at *Z*_tip_ = 20 μm in the area of the yellow box in Fig. 1B. In addition to the clear circular contour, we also note the fluctuating background arising from other nearby TLS outside the grid frame. Within the whole TLS ensemble coupling to the circuit, the resolved contours represent the most strongly coupled and hence most detrimental TLS. They are the dominant source of energy loss and temporal fluctuations [including 1/*f* dielectric noise (*30*)] in qubits and resonators.

The grid data were collected over 3 days, implying that the observed ring and other experimental parameters remained stable throughout. For all presented plots, we subtract a second-order polynomial fit from the measured *S*_21_(*V*_tip_ at each point. This removes the *V*_tip_-independent capacitive contribution from the metallic tip above the resonator and any slow drifts (due to, e.g., thermal relaxation or electrostatic forces). This makes small variations in the signal from local TLS more apparent. Otherwise, they can often be dominated by the capacitive response. Reversing the tip voltage sweep direction does not affect the shape or location of the ring, ruling out any charging effects on the device.

To understand why TLS manifest as rings in our experiment, we consider a TLS with an electric dipole moment $\vec{p}$ interacting with the microwave field ${\vec{E}}_{\text{mw}}$ of a galvanically grounded resonator resulting in a coupling strength $\mathrm{hg}=\vec{p}\cdot {\vec{E}}_{\text{mw}}$. When a sharp AFM tip with a dc voltage *V*_tip_ is positioned at a distance $\vec{r}$ from the TLS, the defect experiences an electric field $\vec{E}\text{dc}\approx V_{\text{tip}}\vec{r}/{|r|}^{2}$. The component, which is aligned with the TLS dipole moment, causes a shift in the TLS energy-level transition frequency

$${\mathrm{hf}}_{\text{TLS}}=\sqrt{\Delta_{0}^{2}+{\left(\epsilon +2\vec{p}\cdot {\vec{E}}_{\text{dc}}\right)}^{2}}$$ (1)

where Δ_0_ is the TLS asymmetry energy and ϵ is an offset energy imposed by the TLS’s local environment (*18*). As the tip voltage is swept, the TLS frequency is tuned along this hyperbolic trajectory (plotted as blue lines in Fig. 2G). When the TLS becomes resonant with the resonator frequency *f*_res_ (black curves in Fig. 2G), it can be detected as a change in the measured *S*_21_ signal. The contours traced out when moving the tip in the *xy*-plane are a set of points where the TLS experiences the same electric field magnitude along the direction of the TLS dipole moment. For the ring shown in Fig. 2, a smaller (larger) tip voltage changes the position $\vec{r}$ at which the tip must be placed for the TLS’s frequency to be shifted to again be resonant with the resonator, and this results in a smaller (larger) ring. This shrinking of the ring with increasing tip voltage is also demonstrated in Fig. 2H, where we show that the radius of the ring in Fig. 2 (A to F) decreases linearly with the applied tip voltage for ring radii ≳*Z*_tip_, as expected from Eq. 1.

As the tip voltage is increased, in most cases, we expect to observe either a shrinking or a growing ring. For very large tip voltages, one could expect to see two concentric contours originating from each of the two intersection points of *f*_res_ with the TLS hyperbola (Fig. 2G). These could be both either shrinking or growing with increasing tip voltage. A third possibility is that one contour is shrinking, followed by one that is growing. These three scenarios depend on at what electric field strength with respect to ground the TLS minima is located.

In the experiment, we kept $|{\vec{E}}_{\text{dc}}|$ below 2 × 10^6^ V/m to prevent damaging the tip or sample and, hence, so far have only observed either shrinking or growing single rings.

### Extracting the TLS dipole orientation

**Fig. 3.**
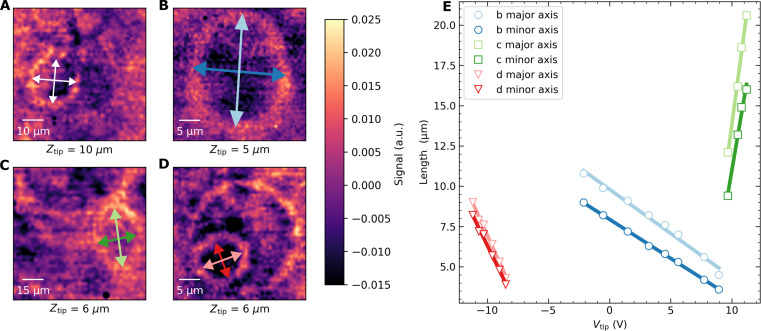
Zooming in on TLS. (**A** and **B**) Close-up images of the TLS ring shown in Fig. 2, taken at (A) *Z*_tip_ = 10 μm and (B) *Z*_tip_ = 5 μm. (**C** and **D**) Two other TLS found elsewhere in the sample, and both images are with *Z*_tip_ = 6 μm. Note the elliptical shape of the contours in (A) to (D), whose minor and major axes (*r*_a_ and *r*_b_, respectively) are marked by double-sided arrows. The contours appear circular for TLS dipole moments pointing mostly perpendicular to the sample surface, such as the large ring in (D). (**E**) Analogous to Fig. 2H, the major and minor axes of all three ellipses shrink or grow linearly with applied tip voltage. The straight lines are linear fits to the data. From the linear fits, we extract their minor/major (*r*_a_/*r*_b_) radius aspect ratios to be *r*_a_/*r*_b_ = 0.95 for (A), 0.8 for (B), 0.8 for (C), and 0.9 for (D).

Zooming in closer to the defect manifesting as the ring in Fig. 2 and bringing the tip closer to the sample surface results in the images in Fig. 3 (A and B) with *Z*_tip_ = 10 μm and *Z*_tip_ = 5 μm, respectively. Notably, when the tip is closer to the surface, the shape transforms from a circular to an elongated contour, which fits well to an ellipse. The major and minor axes of the fitted ellipse both shrink linearly with applied tip voltage as shown in Fig. 3E, and their ratio remains constant within experimental error. Zoomed-in images of a couple of other TLS defects that appear elliptic (located outside the field of view of Fig. 2) are shown in Fig. 3 (C and D) for *Z*_tip_ ≈ 6 μm. The in-plane orientations of all these ellipses differ and do not appear to correlate with any patterned device features. Instead, the elliptic shape is an indication of the TLS dipole moment orientation.

To extract this orientation, we proceed with modeling the expected response. Following (*31*), the transmission *S*_21_(*f*) of the resonator when the tip voltage tunes a TLS to be resonant with it (i.e., for *f*_TLS_ ≃ *f*_res_) can be expressed as

$$|S_{21}\left(f\right)|=\left|1+\frac{\kappa_{c}}{i\left(f-f_{\text{res}}\right)-\kappa +\frac{g^{2}}{i\left(f_{\text{TLS}}-f_{\text{res}}\right)-\gamma_{\text{TLS}}/2}}\right|$$ (2)

where γ_TLS_ is the TLS linewidth and κ = κ_c_ + κ_i_ is the total resonator loss rate, given by the sum of the coupling and internal loss rates, respectively. When the TLS-resonator coupling *g* is weak (≲100 kHz), the resonator response is mainly dissipative. In contrast, a large *g* also induces shifts in the resonator frequency. Our simulations indicate that, experimentally, we more frequently encounter the former regime than the latter (an example of which is shown in Fig. 1F).

**Fig. 4.**
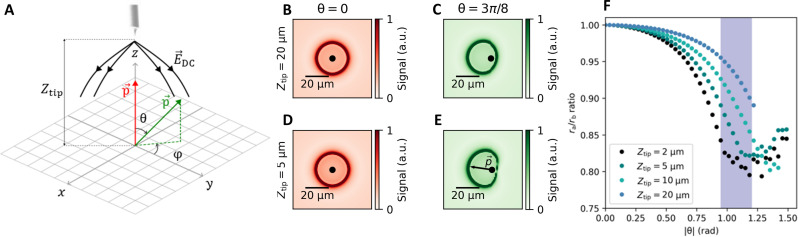
Extracting TLS dipole moment orientations from simulations. (**A**) Schematic of the system used for simulations. (**B** to **E**) Simulated SGM images for TLS on the substrate surface with two different polar angles and two different tip heights using a simplified geometry (see the Supplementary Materials for details). The color scale indicates the expected response in the measured signal analogously to experimental grids. When the dipole moment is oriented perpendicular to the sample plane [red arrow in (A)], the observed rings are always circular. The rings become elliptical when the TLS dipole is oriented to have a large in-plane component [green arrow in (A)] and when the tip is brought close to the surface. The black dots indicate the location of the TLS, and the arrow in (E) indicates the simulated orientation of the in-plane dipole moment. (**F**) Simulations showing how the aspect ratio of ellipses varies with the tip-sample distance and polar angle θ. For each data point, the tip voltage has been adjusted such as to produce contours with the same *r*_b_ ≈ 18.5 μm, a scale close to the observed ellipses in Fig. 3. For all the simulations, we use experimentally consistent parameters of γ_TLS_ = 150 MHz, *g* = 100 kHz, *f*_readout_ − *f*_res_ = 20 kHz, and κ = 100 kHz.

Information about the orientation of the TLS dipole moment is implicitly present in Eq. 2 via *f*_TLS_, defined in Eq. 1. Here, the term $\vec{p}\cdot {\vec{E}}_{\text{dc}}$ implies that we can be selectively sensitive to the in-plane or out-of-plane component of $\vec{p}$ by changing the direction of the applied electric field ${\vec{E}}_{\text{dc}}$, enabled by a sharp tip. In Fig. 4A, we define the TLS orientation by the in-plane (ϕ) and out-of-plane (θ) angles with respect to the tip coordinate system.

For a tip with a set voltage held at a particular height *Z*_tip_ above the sample, the electric field distribution ${\vec{E}}_{\text{dc}}$ on the sample surface is computed in COMSOL Multiphysics software. For a chosen orientation of the TLS dipole moment $\vec{p}$, the spatial ${\vec{E}}_{\text{dc}}$ data are then used to calculate *S*_21_ from Eq. 2. In Fig. 4B, for illustration, we show these simulated TLS images for two different orientations of a TLS dipole moment for two different *Z*_tip_. This shows the expected circular contour for θ = 0 for both tip heights as the *z* component of the electric field dominates in both cases. However, for large θ and small *Z*_tip_, the in-plane component of the electric field becomes increasingly important and an elliptical contour emerges. From this elliptical contour, the in-plane angle ϕ of the TLS dipole moment can be inferred directly from the angle of the short axis of the contour, meaning we can directly read ϕ from the panels in Fig. 3 (A to D).

Deducing the polar angle θ requires more detailed simulations. We fit the simulated two-dimensional *S*_21_ map (similar to images in Fig. 4, C and E) to elliptical contours to extract the major (*r*_a_) and minor (*r*_b_) axes of the ellipses. The process is repeated as a function of θ and *Z*_tip_ to obtain Fig. 4F. The plot shows that the change in *Z*_tip_ from 10 to 5 μm and resulting contour aspect ratio experimentally seen in Fig. 3 (A and B) corresponds to a narrow (shaded) range of possible θ for this TLS. Also, note is that the smallest possible *r*_a_/*r*_b_ ratio obtainable is 0.8 for large θ, in good agreement with experimental observations as we have not seen any TLS with a smaller ratio. We note that in Fig. 4F, for very large θ, the *r*_a_/*r*_b_ ratio again starts to increase. This is due to the resonance contour starting to deviate from an elliptical shape.

The simulations indicate that for large θ the actual defect is located slightly off-center along the minor axis of the ellipse. Furthermore, they suggest that both the center of the ellipse and its aspect ratio change slightly with the applied tip voltage. Unfortunately, the large fields of view required to visualize these transitions are beyond the scan range of our microscope.

Although the slopes in Figs. 2H and 3E indicate the relative magnitudes of the TLS dipole moments, uncertainty in the exact electric field at the defect, and the unknown ϵ of each defect prevents extraction of $|\vec{p}|$. However, to reproduce the experimental results in our modeling, we assume $|\vec{p}|=1$ eÅ (*18*, *32*, *33*), which results in tip voltages very close to those used in our experiments. To precisely extract ∣*p*∣, simultaneous frequency tuning of the device is required (*18*).

## DISCUSSION

Of the multitude of TLS present in the sample, our experimental setup is most sensitive to only those defects that are near the surface, close to the resonator, and thus couple strongly to it. Thus, the observed TLS ought to be those most debilitating to device coherence.

Several factors can affect the shape of the observed contours. The width of the contour is related to the quality factor of the resonator, the linewidth of TLS, and ∂*f*_TLS_/∂*r*. The spatial resolution is also limited by the sharpness of the AFM tip. Scanning electron microscope images of the tip, captured before and after the experiment (see the Supplementary Materials for details), demonstrate that the tip apex was never larger than a few microns during the 6-month experiment, setting our resolution. The out-of-plane tilting of the sample can also distort the contours. However, the ~2° tilt of the sample measured using AFM should result in a tip-sample height difference of only ≈700 nm over a 20-μm scan range, resulting in minimal distortion.

Another factor blurring the experimental images at small *Z*_tip_ is the mechanical vibrations of the tip. Taking grid measurements with very small tip-sample distances were difficult, likely due to these mechanical vibrations in our system coming from the pulse tube cryocooler. In particular, attempting a grid over the defect in Fig. 3B at *Z*_tip_ = 1 μm resulted in the TLS ring disappearing also in subsequent scans at larger *Z*_tip_. We speculate this to be because of accidental contact with the defect, potentially also demonstrating the delicate glassy state of the TLS, susceptible to even minute perturbations. More stable scanning will allow closer imaging and improved resolution down to tens of nanometers, ultimately limited by the achievable in-plane electric field gradient from the tip, set by the tip size.

Future experiments will undoubtedly have better control over these aspects, leading to a more precise determination of θ. Our simulations show that as long as the TLS defect is located a sufficiently large distance from any metalisation on the sample, the specific device geometry does not play a substantial role. For TLS located very close to metallic structures on the sample (few nanometers, for example, in the metal oxide), however, the electric fields will always be perpendicular to the metal, and hence, these TLSs will always appear as circular contours. It also means that the TLS observed here as ellipses are located on or in the dielectric substrate (within the gap of the interdigitated capacitor of the resonator).

Several improvements to our experiment can yield substantially more information about the defects. For example, using a substrate back gate and applying an additional variable electric field in the vertical direction can pinpoint the location of defects in three dimensions. Recent studies have attributed most of the decoherence in superconducting devices to surface losses (*15*, *34*, *35*), and a systematic study of TLS concentrations with height would be very beneficial to this discussion.

The bulk of our knowledge about TLS in superconducting quantum circuits is based on phenomenological models deduced from observing the behavior of the devices they inhabit. Direct interrogation of individual defects is rare, and because of the complexity of the setup required, relatively little attention has been focused on understanding the physical and chemical nature of these defects. Our approach of combining scanning probe systems with live quantum circuit readout is a promising direction to directly investigate the assumptions underpinning our current understanding of decoherence in quantum circuits. Localizing the defects using our technique will also facilitate their study using other established scanning probe and surface analysis techniques. Studying multiple devices and different fabrication techniques can help generate statistics on the concentration and origins of TLS in different materials. These experimental approaches, coupled with atomistic modeling (*36*–*39*), can aid in the understanding and eventual mitigation of TLS defects.

## MATERIALS AND METHODS

The NbN on Sapphire resonator sample used in this work consists of a 3λ/4 hanger-type resonator. It consist of two parallel prongs that serve as inductors, connected together on one side of the resonator. A series of interdigitated capacitors couple the two prongs to each other. The interdigitated capacitors concentrate the electric fields inside the resonator, thereby enabling stronger coupling to TLS, facilitating easier detection. The two prongs are connected to the ground plane on either side of the resonator (through an inductive filter, seen in the scan of Fig. 1C) to facilitate the application of a dc current through the resonator. This can be used to tune the resonance frequency, a property not used in this work.

The 3λ/4 fundamental mode of the resonator results in the microwave voltage standing wave amplitude as sketched in Fig. 1B. The inductive filters are located in the voltage node to maximize the quality factor of the resonator. In this region, the sensitivity to TLS is reduced as here they couple weakly to the resonator microwave field. Instead, we detect the TLS most detrimental to device performance near the voltage anti-nodes.

For more details of the experimental setup, please refer to the Supplementary Materials.
