# Supplementary material for: In situ scanning gate imaging of individual quantum two-level system defects in live superconducting circuits
Source: Sci Adv. 2025 Apr 30;11(18):eadt8586. doi: 10.1126/sciadv.adt8586 (PMC12042869; doi:10.1126/sciadv.adt8586)
Supplement: Supplementary file 1 — Supplementary Text Figs. S1 to S9 Legend for movie S1 [file sciadv.adt8586_sm.pdf]

Science Advances
AAAS

Supplementary Materials for

In situ scanning gate imaging of individual quantum two-level system defects in live superconducting circuits

Marius Hegedüs *et al.*

Corresponding author: Riju Banerjee, riju.banerjee@npl.co.uk; Sebastian E. de Graaf, sdg@npl.co.uk

Sci. Adv. **11**, eadt8586 (2025)
DOI: 10.1126/sciadv.adt8586

The PDF file includes:

Supplementary Text
Figs. S1 to S9
Legend for movie S1

Other Supplementary Material for this manuscript includes the following:

Movie S1

1. EXPERIMENTAL MICROWAVE SETUP

Supplementary Fig. **S1** shows the configuration of the microwave wiring inside the dilution refrigerator, and the setup of the heterodyne detection scheme at room temperature. In brief, two low-frequency (30 MHz) phase-shifted signals are up-converted to a single side-band tone at the sample resonance frequency, and passed down a heavily attenuated coaxial line to the sample on the SPM stage. The signal from the sample is then returned via a travelling-wave parametric amplifier (TWPA, Silent-Waves Argo; driven by a pump tone at $f_P \approx 6$ GHz) before being further amplified by a high-electron mobility transistor (HEMT) amplifier at the 4K stage of the cryostat, and further amplified at room temperature to the desired level. The signal is then again down-converted to 30 MHz and demodulated using a lockin-amplifier, which feeds the two analog demodulated quadrature signals, proportional to the microwave transmission at the chosen frequency $S_{21}(f)$, to the SPM control electronics (Nanonis). We record the data from both quadratures, but rotate the phase such as to put most of the signal in one of the quadratures. For simplicity, the data shown in the manuscript is from one of these quadratures only.

2. DEVICE LAYOUT

In Supplementary Fig. **S2** we show details of the microwave resonator layout. The resonator consists of two prongs connected together at one end (bottom) and forming an interdigitated capacitor where the microwave electric field strength is concentrated in-between the prongs. The two open ends of the resonator are connected via a coupling capacitor to the transmission line and ground respectively.

Supplementary Fig. **S2**(b) shows the simulated charge density at the resonance frequency of the microwave resonator used in this experiment. Simulations were carried out using Sonnet. The calculated charge density follows the expected distribution for a $3\lambda/4$-mode. The charge density is localised in-between the two prongs of the interdigitated capacitor of the resonator, meaning the microwave electric field is strongly localised to this region.

Supplementary Fig. **S2**(c) shows the equivalent circuit schematic of the resonator, together with an optical image of a zoomed in region on the resonator, showing the same pattern as in the obtained AFM scan in Fig 1 of the main manuscript. At the voltage (charge density) node each prong is galvanically connected to ground via an inductor L_g. This ensures the whole resonator structure is grounded, as a reference potential for the external electric field applied by the tip.

3. SEM IMAGES OF THE TIP

The tip used for AFM and applying the local gate voltage was produced by etching a 0.25 mm tungsten wire in a KNO solution. The etched tip was cleaned in deionised water to both stop the etching process and clean any residual salts sticking to it. The tip was then imaged using SEM to verify its sharpness. An SEM image of the tip taken before scanning is shown in Supplementary Fig. **S3**a.

Images taken after scanning for six months (Supplementary Fig. **S3** b and c) show that it became blunt over time. Nevertheless, the end diameter was still less than 5 μm. The simulation results presented in this work used a tip with a diameter of $\lesssim 5$ μm to accurately model our observations.

4. TLS SATURATION AND POWER DEPENDENCE

A common signature of TLS is through their power dependence. At low microwave powers, TLS can absorb photons from the resonator, making TLS the primary source of loss in the circuit. At higher microwave powers, TLS cannot dissipate the absorbed energy as phonons quickly enough, causing them to saturate. In Supplementary Fig. **S4** we show the power dependence of the internal and external (coupling) quality factors of the resonator in which TLS were imaged in this work.

We estimate the average photon number by $\langle N \rangle = Q^2 P_{\text{in}}/(8\pi Q_{\text{ext}} \hbar f_0^2)$, where Q is the total and Q_{ext} the external (coupling) quality factors obtained from fits to the S_{21} VNA data, and P_{in} is the microwave power reaching the sample. In Fig. **S4** we show the internal and external quality factor as a function of $\langle N \rangle$. The data is fitted to $Q_{\text{int}}^{-1} = F \tan\delta/(1 + \langle N \rangle/N_c)^\alpha + Q_{\text{int},0}^{-1}$, finding $\alpha = 0.406 \pm 0.04$, critical photon number $N_c = 13 \pm 7$, TLS limited loss $F \tan\delta = (4.1 \pm 0.6) \times 10^{-6}$ and power-independent loss of $Q_{\text{int},0} = (1.8 \pm 0.2) \times 10^6$. The quoted error bounds include propagated errors from the Q_{int} data. This strong dependence of Q_{int} on power indicates that the quality factor is strongly limited by TLS, which are saturated at increased driving powers.

We further confirm TLS saturation by imaging individual TLS in our SGM setup. This is shown in Supplementary Fig. **S5**. For our experiments, we found that an average photon number in the range of $\langle N \rangle \approx 100 - 1000$ provides a good compromise between TLS sensitivity and signal-to-noise ratio.

Supplementary Fig. **S5**, shows grids taken at the exact same location at different driving powers, by adjusting the variable attenuator on the signal input line shown in Supplementary Fig. **S1**. From each panel, a background has been subtracted and all are plotted in the same colour scale. At low powers (high attenuation, top panels), we see a ring that grows with increasing tip voltage. The fluctuations reduce and ultimately disappear as the driv-

ing power is increased (bottom panels), showing that this individual TLS is saturated.

5. ELECTROSTATICS MODELLING

To simulate the frequency shift of a TLS and the resultant change in the S_{21} transmission as it becomes resonant with the resonator, a simplified tip and sample geometry was simulated. In particular, a conical tip with a hemispherical bottom was chosen for the tip geometry (Supplementary Fig. **S6**). The dimensions of the tip were chosen to be comparable to that of the actual tip dimensions, as measured by SEM imaging (in Supplementary Fig. **S3**). A square 100 μm wide and 4 μm thick substrate slab of sapphire under the tip imitated the sample. The underside of the sapphire slab was grounded and the tip was held at a potential of 1 V. While this is a substantial oversimplification of the sample geometry, the agreement between experimental and simulated results shows that our technique is able to capture the TLS dipole orientation even in the simplest of scenarios. A number of unknown experimental parameters (e.g. exact tip size and shape, exact local electric field strength, TLS location within the substrate/surface) will influence the exact determination of θ. Future studies will undoubtedly have increased knowledge of these parameters.

Supplementary Fig. **S6** shows the magnitude of the simulated electric fields $\vec{E}_{DC}$ in the vertical (E_z) and horizontal (E_y) directions around the tip.

Similarly, Supplementary Fig. **S7** (a-b) shows the resulting electric field strength in the sample plane, separated into the Z and X components respectively.

Supplementary Fig. **S7** (c-d) compares the same electric field strengths as in Supplementary Fig. **S7** (a-b) at $Y = 0$ for different tip-sample separations for (c) E_z and (d) E_x. Here we have scaled the data by the expected $E \propto 1/Z$ scaling. For E_z we see that this almost collapses the curves at $x = 0$ (some deviation due to finite tip size), and the larger Z_{tip} results in a more delocalised electric field distribution, as expected. In Supplementary Fig. **S7** (d) we show the behaviour of E_x with the same scaling applied. Here we see similar broadening, and we also see that the lateral component of the electric field vanishes much faster with increased tip-sample distance. I.e. ellipses could only be observed for small Z_{tip}.

To mimic the change in voltage on the tip in the experiment we multiply the resulting E_{DC} with a prefactor, before calculating the measured signal quantity through Eq. (2). Varying the tip voltage in simulation reproduces the change in size of the rings. As an example, in Supplementary Fig. **S8** we plot the ring radius as a function of tip voltage, using parameters for the TLS resulting in a ring similar to that in Fig. 2 of the main text. Also, in simulations the ring radius shrinks approximately linearly with applied tip voltage, in analogy with Fig. 2h.

6. DATA ANALYSIS

As the tip moves in a grid pattern, the measured $|S_{21}(f_{\text{readout}})|$ vs V_{tip} at each point is a combination of the added capacitance of the tip on the resonator, small thermal drifts of the resonator arising from tip motion, along with the local fluctuations from the TLS of interest. For each point on the grid, the recorded $S_{21}(V_{tip})$ was fitted to a second order polynomial that was then subtracted from the raw data. The linear and quadratic term accounts for small thermal drifts (and possible electrostatic forces for small tip-sample distances), and the constant offset constitutes the capacitive background (which is independent of V_{tip}). By subtracting this we are left with only the part that has a fast variation in V_{tip}, i.e., the specific defects that are momentarily tuned into resonance.

As an example of the capacitive background we refer to the Supplementary Fig. **S9**a, where the raw data of the panel in Fig. 3a of the main text is plotted. In Supplementary Fig. **S9**b we show an example of this parabolic subtraction at one point in the grid marked by a star in Supplementary Fig. **S9**a.

7. MOVIE OF THE DATASET IN FIG. 2

The Supplementary movie S1 containing the full dataset presented in Fig. 2 of the main text is presented in the file 'Movie S1'.

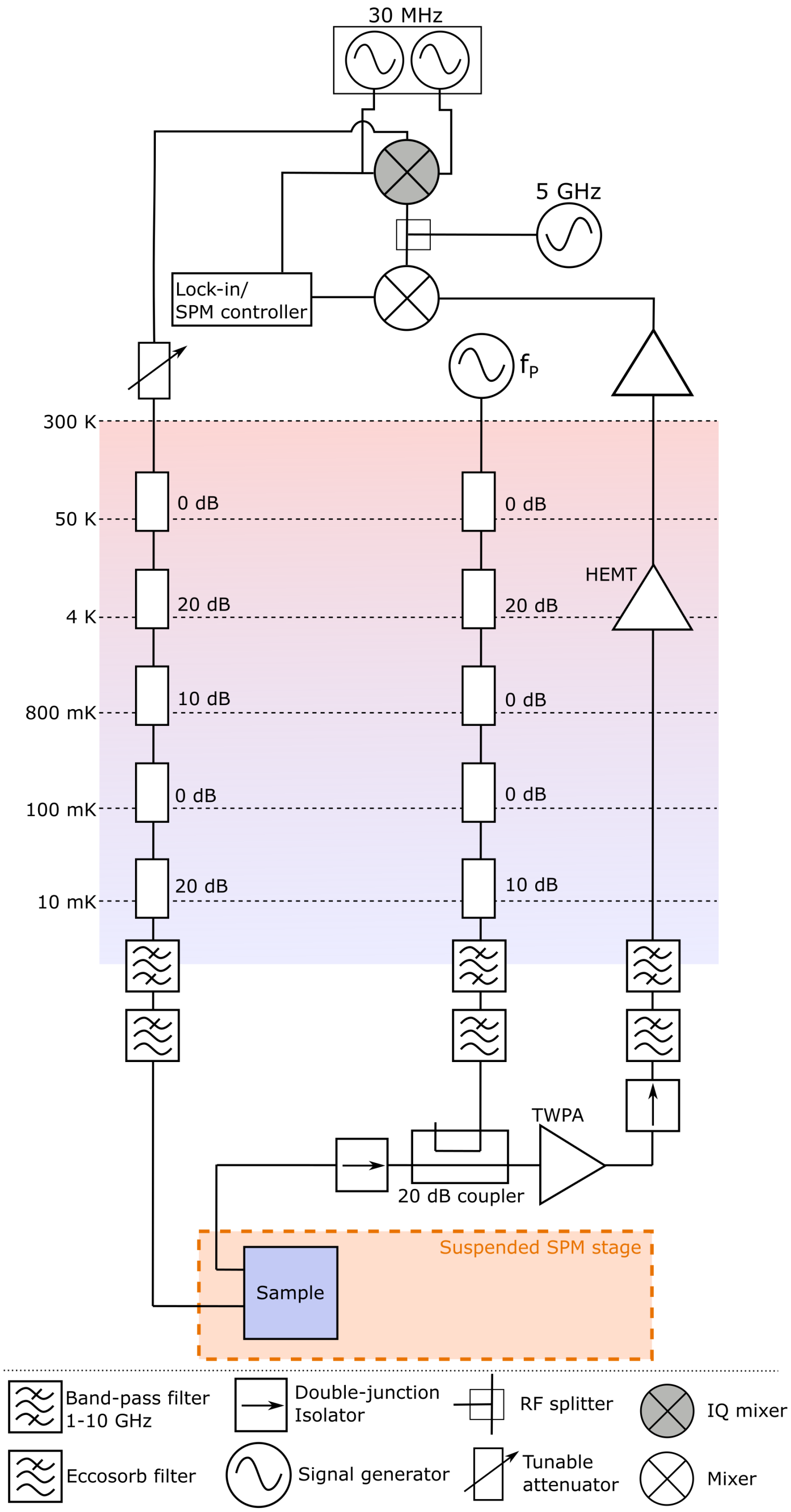

Supplementary Fig. S1: Heterodyne measurement setup and fridge RF wiring.

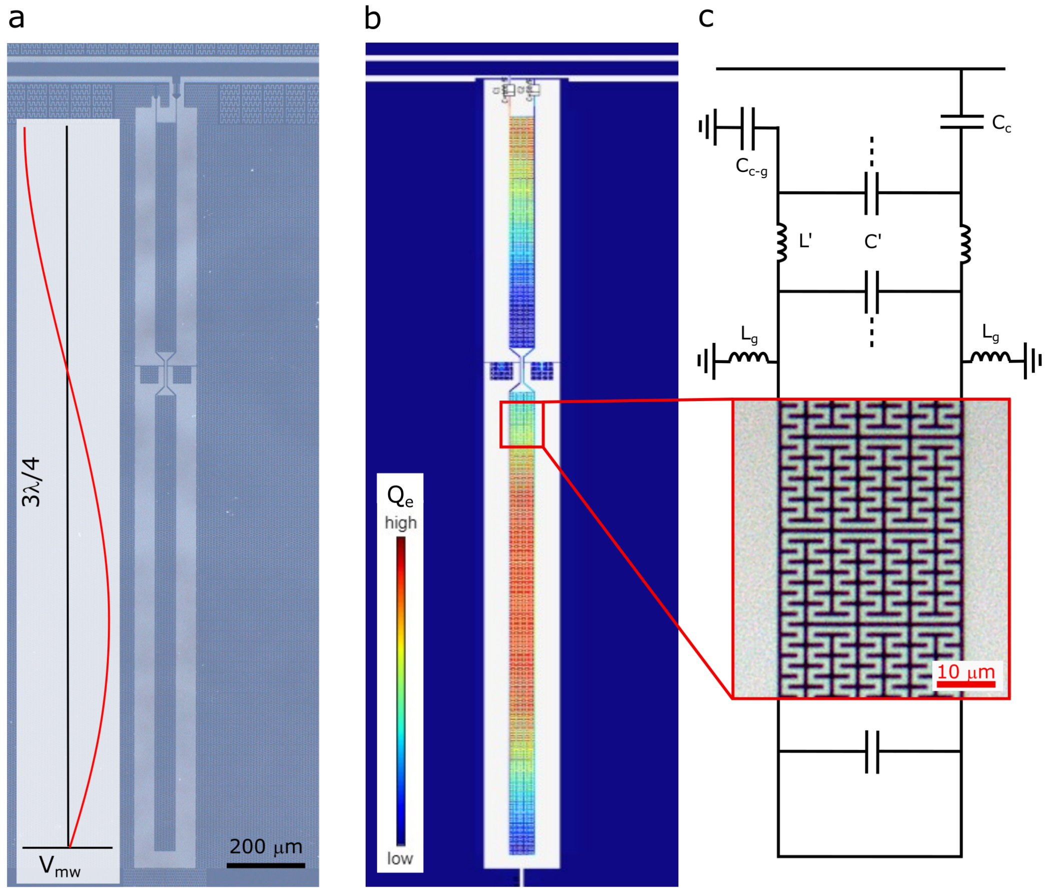

Supplementary Fig. S2: **Details of the sample layout.** (a) optical image of the sample (same as in Figure 1 in the manuscript). (b) Sonnet simulation of the same structure showing the charge density Q_e at the resonance frequency. (c) Equivalent circuit schematic of the resonator, having a per unit length capacitance C' and inductance L'. One prong is coupled with a coupling capacitance C_c to the transmission line, and the other prong is coupled to ground with a capacitance C_{c-g}. At the voltage (charge density) note each prong is galvanically connected to ground via an inductor L_g. The image shows a zoomed in region of the interdigitated capacitor forming the resonator, where bright contrast is the substrate (sapphire) and dark contrast superconductor (NbN).

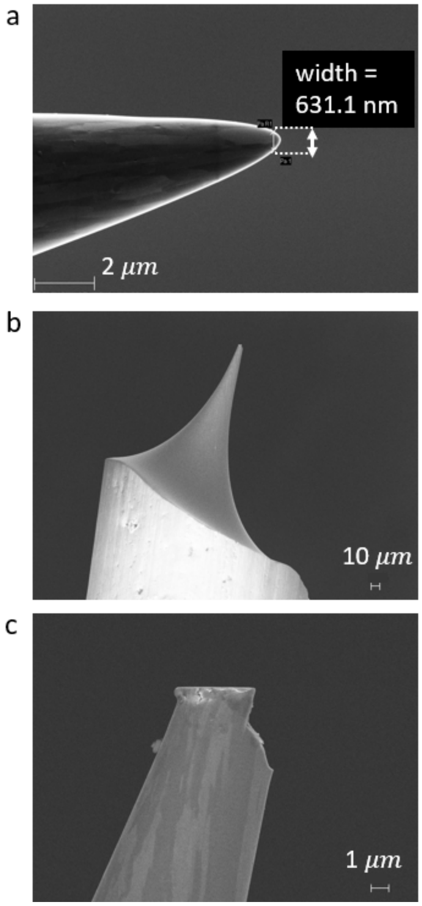

Supplementary Fig. S3: **SEM images of the tip.** (a) SEM image of the tip taken before scanning shows a sharp (sub-micron) tip. Large area (b) and zoomed-in (c) SEM images of the tip taken after scanning. The end is blunted due to scanning for 6 months, but is still less than 5 microns wide.

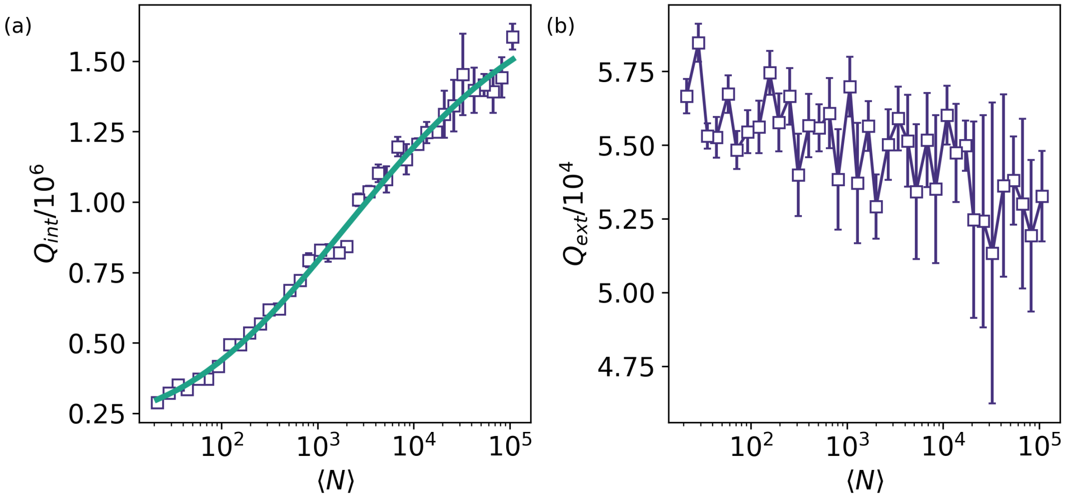

Supplementary Fig. S4: **Quality factor of the resonator sample measured on the scanning gate microscope sample platform.** (a) Internal quality factor at different average photon occupancy. The solid line is a fit to the standard tunneling model (see text). (b) The external quality factor as a function of average photon number. Data taken at a temperature of 45 mK. Error bars are 95% confidence bounds from fits.

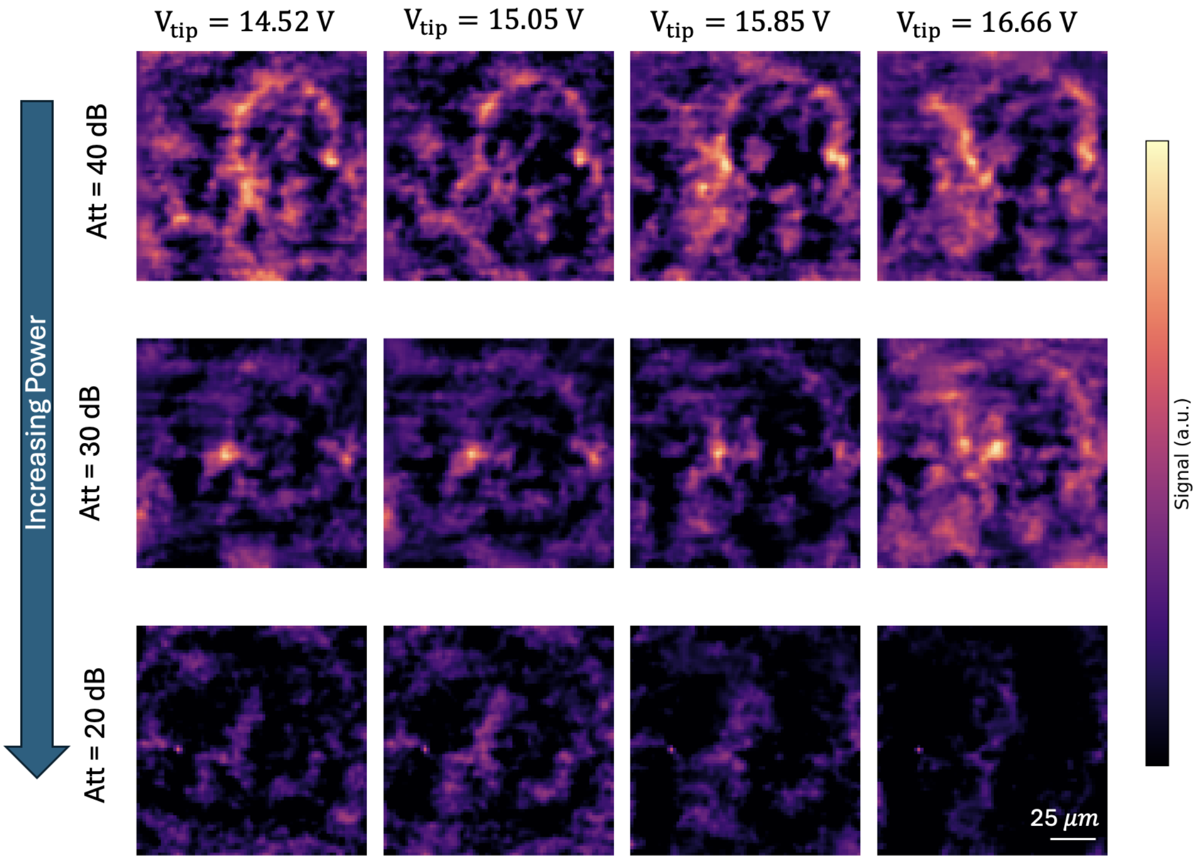

Supplementary Fig. S5: **Power dependence of TLS.** Grids taken at the exact same location at low power (top panels) show more more pronounced TLS contours than those at larger powers (bottom panels). All panels are plotted on the same colour scale and a fitted background has been subtracted to highlight the fluctuations. All three grids were taken with the tip 15 μm above the sample. The scale bar in the bottom right is shared across all panels.

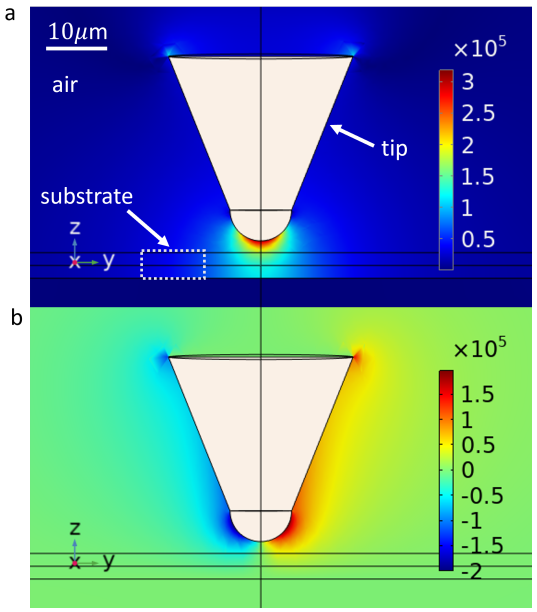

Supplementary Fig. S6: **Simulated tip electric field.** (a) Tip Electric field in the z-direction $|E_z|$, (b) Electric field in the y-direction. Color scale is electric field strength in units of V/m.

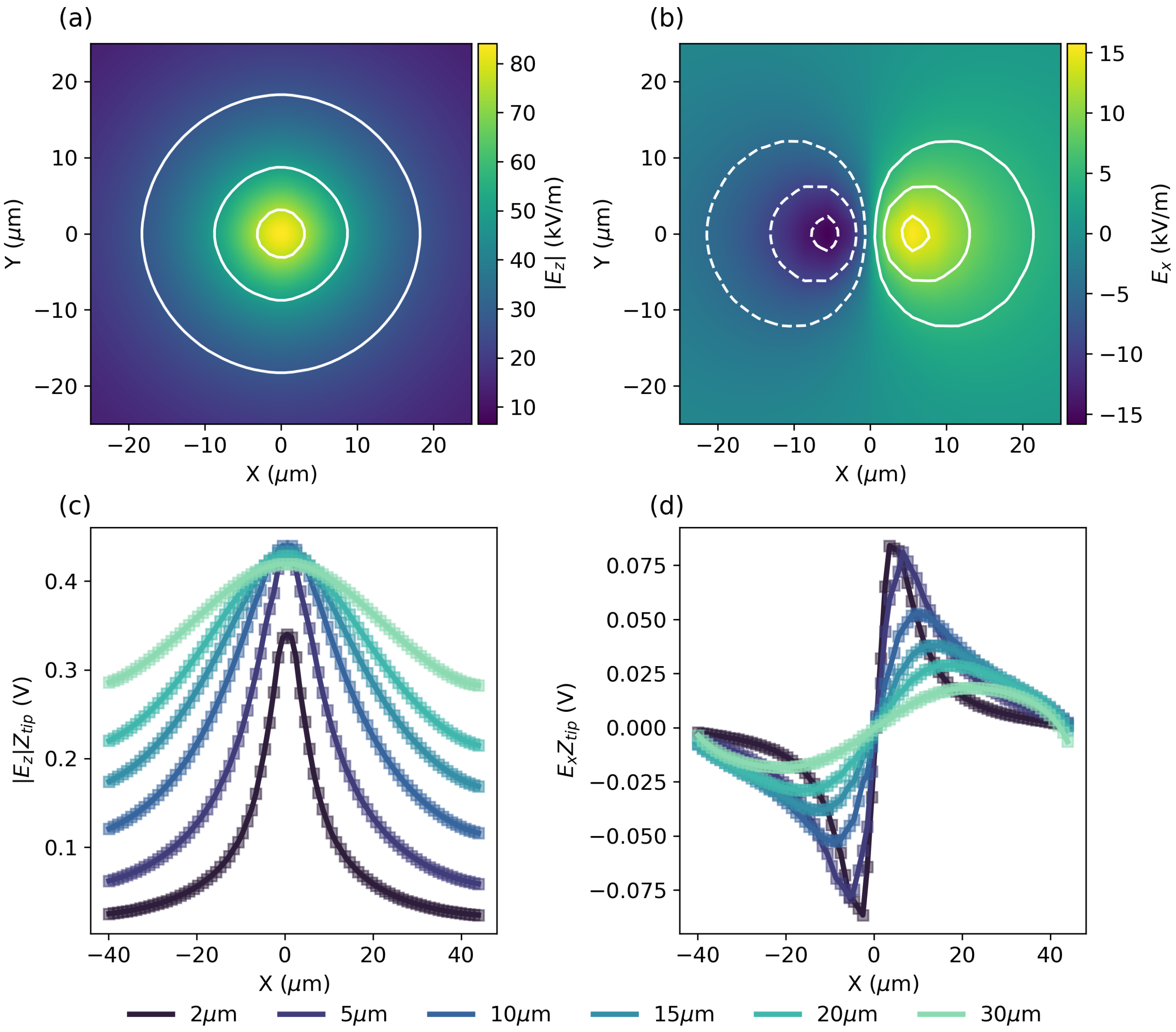

Supplementary Fig. S7: **Simulated tip electric fields at the sample plane.** (a-b) Simulated electric field strengths in the sample plane for $Z_{\text{tip}} = 5$ μm. (c-d) Cross-sections of simulated data similar to (a) and (b) for different Z_{tip} (different colors), normalised by the tip-sample distance $1/Z_{\text{tip}}$.

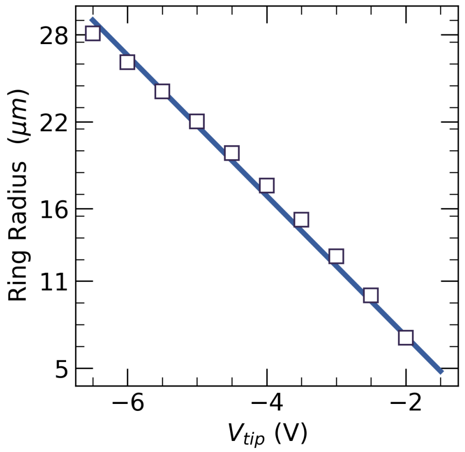

Supplementary Fig. S8: **Simulated ring radius as a function of applied tip voltage.** A linear fit (solid line) to the ring radii for varying simulated tip voltages (markers). A dipole orientation of $\theta, \phi = 0$ was used. There is a clear linear dependence of the radius with voltage, as observed experimentally.

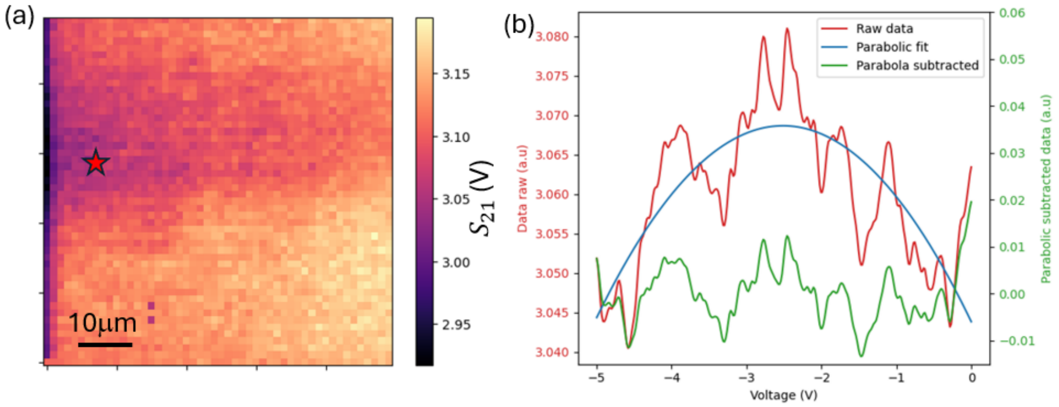

Supplementary Fig. S9: **Subtracting the background signal** (a) Raw dataset for the image in Fig. 3a of the main text for $V_{tip} = -4.31V$, same as that of figure 3a. The color scale is the raw voltage from the heterodyne measurement setup sampled by the scanner controller. (b) Signal vs tip voltage taken at the point marked by the red star in the left panel showing the raw data, the fitted parabola (left scale), and the data with the fit subtracted (scale on the right).

Supplementary movie S1. Full dataset of Fig. 2 The full dataset of the panels shown in Fig 2 (a)-(f) of the main text is presented in the accompanying file, titled 'Movie S1'.
